# Scutellarin Alleviates Zearalenone-Induced Injury in Porcine Ovarian Granulosa Cells Through WNT5A-Associated Regulation of Cell-Cycle-Related Proteins

**DOI:** 10.3390/vetsci13070719

**Published:** 2026-07-22

**Authors:** Hua Zhang, Wenwen Ding, Yanyan Yi, Xinyue Zhang, Panpan Sun, Kuohai Fan, Wei Yin, Huizhen Yang, Zhenbiao Zhang, Jia Zhong, Yaogui Sun, Jianzhong Wang, Shaoyu Wang, Hongquan Li, Na Sun

**Affiliations:** 1 Shanxi Key Laboratory for Modernization of TCVM, College of Veterinary Medicine, Shanxi Agricultural University, Jinzhong 030801, China; zhsn060511@126.com (H.Z.); dww1030@126.com (W.D.); zhang623422@163.com (X.Z.); sunpp0505@163.com (P.S.); dkyyinwei@126.com (W.Y.); hzyang2020@163.com (H.Y.); zbzhangvet@sxau.edu.cn (Z.Z.); zhongjia3294@163.com (J.Z.); dkyypb@163.com (Y.S.); jianzhongwang@cau.edu.cn (J.W.); livets@163.com (H.L.); 2 The Youth Innovation Team of Shaanxi Universities, Shaanxi A&F Technology University, Yangling 712100, China; yieryan2015@163.com; 3 Laboratory Animal Center, Shanxi Agricultural University, Jinzhong 030801, China; fkhyxj@163.com; 4 School of Dentistry and Medical Sciences, Charles Sturt University, Orange, NSW 2800, Australia; shawang@csu.edu.au

**Keywords:** scutellarin, zearalenone, ovarian granulosa cells, cell cycle, WNT5A

## Abstract

Zearalenone is a common mycotoxin that impairs reproductive function, particularly by damaging ovarian granulosa cells. This study investigated the protective effect and underlying mechanism of scutellarin against zearalenone-induced injury in porcine ovarian granulosa cells. Cells were isolated and identified by follicle-stimulating hormone receptor immunofluorescence. Cell viability, cell-cycle distribution, and related molecular changes were evaluated using the MTT assay, flow cytometry, qRT-PCR, and Western blot. Scutellarin showed no obvious cytotoxicity within the tested range and attenuated the zearalenone-induced reduction in cell viability. Flow cytometry analysis demonstrated that scutellarin alleviated zearalenone-induced cell-cycle disturbance. At the molecular level, scutellarin upregulated the expression of cell-cycle-related factors, including CDK1, CDK2, CDK4, and PCNA, and increased the expression of Wnt/β-catenin signaling-related proteins, including WNT5A, β-catenin, c-MYC, and CCND1. WNT5A knockdown further indicated that scutellarin-mediated regulation of PCNA, CDK1, and CDK4 is WNT5A-dependent, whereas its effect on CDK2 may involve a WNT5A-independent mechanism. These findings indicate that scutellarin alleviates zearalenone-induced granulosa cell injury partly through WNT5A-associated modulation of cell-cycle-related proteins, providing mechanistic insights for its protective effects.

## Simple Summary

Zearalenone is a fungal toxin that can contaminate animal feed and impair reproduction, partly by injuring ovarian cells. This study examined whether scutellarin, a natural compound found in plants, could protect pig ovarian cells from this type of damage. The cells were exposed to zearalenone with or without scutellarin, and we assessed their survival, growth pattern, and changes in proteins related to cell division. Zearalenone reduced cell survival and disturbed the normal process of cell growth and division. Scutellarin was not harmful to the cells at the tested concentrations and lessened the damage caused by zearalenone. It also helped restore the normal regulation of cell division. Further results suggested that scutellarin worked partly through a cell communication pathway that controls cell growth. These findings indicate that scutellarin may help protect ovarian cells from zearalenone-induced injury and provide preliminary experimental evidence for further evaluating scutellarin as a potential protective agent against zearalenone-related reproductive damage.

## 1. Introduction

Zearalenone (ZEA) is a prevalent mycotoxin worldwide. It is a non-steroidal estrogenic toxin mainly produced by Fusarium species, including *F. graminearum*, *F. roseum*, and *F. moniliforme* [1,2]. Because of its high stability in common feed ingredients, such as corn, grains, and oilseeds, ZEA represents an important source of mycotoxin contamination in animal feed [3,4]. ZEA has a chemical structure similar to that of endogenous mammalian estrogens and can competitively bind to estrogen receptors, thereby exerting estrogen-like effects and potentially causing estrogenic syndrome [5]. In reproductive tissues, ZEA exposure has been associated with abnormal uterine and ovarian development, including endometrial hyperplasia, premature follicular development, and follicular atresia [6]. Among animal species, pigs are particularly sensitive to the estrogenic effect of ZEA [7,8]. Previous studies have shown that ZEA induces apoptosis in porcine ovarian granulosa cells [9,10]. ZEA exposure for 24 h also inhibits cell proliferation, induces DNA damage, disrupts genomic stability, and suppresses granulosa cell growth [11]. Chen et al. systematically reviewed the physicochemical properties, toxic effects, and mechanisms of ZEA-induced reproductive damage, providing an important reference for studies on interventions against ZEA toxicity [12].

In recent years, several traditional Chinese medicines and their active components have been reported to alleviate ZEA-induced reproductive toxicity. For example, proanthocyanidins inhibit ZEA-induced apoptosis and oxidative stress in porcine testicular cells through activation of the NRF2 signaling pathway [13]. Isorhamnetin protects porcine ovarian granulosa cells from ZEA-induced damage through the PI3K/Akt signaling pathway, and curcumin pretreatment ameliorates ZEA-induced disruption of redox homeostasis in porcine ovarian granulosa cells [14,15]. These findings suggest that natural compounds may provide potential strategies for reducing ZEA-induced reproductive toxicity.

Yi et al. previously showed that scutellarin inhibits ZEA-induced apoptosis in mouse ovarian granulosa cells through the mitochondrial apoptosis pathway [16]. Our subsequent studies integrating network pharmacology, target fishing, molecular docking, and surface plasmon resonance (SPR) analysis suggested that WNT5A may be a potential target involved in the protective effect of scutellarin in granulosa cells [17,18]. However, the role of WNT5A-associated cell-cycle regulation in the effect of scutellarin against ZEA-induced injury in porcine ovarian granulosa cells remains unclear. Therefore, the present study investigated whether scutellarin alleviates ZEA-induced injury in porcine ovarian granulosa cells by regulating cell-cycle progression and WNT5A-associated signaling, thereby providing a mechanistic basis for its potential application in protecting ovarian granulosa cells from mycotoxin-induced damage.

## 2. Materials and Methods

### 2.1. Isolation and Culture of Porcine Ovarian Granulosa Cells

Porcine ovarian granulosa cells were isolated using a previously described method with minor modifications [19]. Porcine ovaries were obtained from the slaughter workshop of Kaiyuan Meat Co., Ltd., Jinzhong, China. The ovaries were collected and transported to the laboratory in prewarmed PBS at 37 °C. After being washed with 75% ethanol and sterile PBS, the ovaries were transferred to a sterile cell culture room. Follicular fluid was aspirated using a 2.5 mL syringe, filtered through a 200-mesh sterile cell strainer into sterile centrifuge tubes, and centrifuged at 100 g for 6 min. This washing and centrifugation step was repeated twice. After the supernatant was discarded, the cells were resuspended in DMEM/F-12(HAM) 1:1 medium supplemented with 10% FBS (Biological Industries, Beit Haemek, Israel). The cells were then seeded into cell culture dishes at a density of 1 × 10^6^ cells/mL and incubated at 37 °C in a humidified atmosphere containing 5% CO_2_.

### 2.2. Identification of Porcine Ovarian Granulosa Cells

The follicle-stimulating hormone receptor (FSHR), a commonly used marker of granulosa cells, was used to identify the isolated porcine ovarian granulosa cells [20]. Porcine ovarian granulosa cells were seeded into 15 mm confocal laser culture dishes (NEST, Wuxi, China) and cultured until they reached approximately 70% confluence. The cells were fixed with anhydrous ethanol for 20 min, permeabilized with 0.2% Triton X-100 (Solarbio, Beijing, China) for 15 min, and blocked with 3% BSA (Solarbio, Beijing, China) for 20 min. For the test group, the cells were incubated with an anti-FSHR antibody (Proteintech, Wuhan, China, 1:50) at 37 °C for 2 h. For the control group, PBS was used instead of the primary antibody. The cells were then incubated with Alexa Fluor 488-conjugated AffiniPure Goat Anti-Rabbit IgG (H + L) (Proteintech, Wuhan, China, 1:100) at 37 °C for 1 h in the dark. Subsequently, the nuclei were stained with DAPI for 3 min. After the DAPI solution was removed, 1 mL of PBS was added to each dish, and the cells were observed using a confocal laser scanning microscope (Leica Microsystems, Wetzlar, Germany). Porcine ovarian granulosa cells were independently isolated from three pigs and used as three biological replicates (*n* = 3).

### 2.3. Scutellarin Treatment

Porcine ovarian granulosa cells were seeded into 96-well plates at a density of 1 × 10^5^ cells per well and cultured until confluence exceeded 90%. Scutellarin was purchased from the National Institutes for Food and Drug Control (Beijing, China; catalog No. 110842-202010; purity, 91.5%). Based on our previous studies of ZEA-induced injury in mouse ovarian granulosa cells [21], 2000 μg·mL^−1^ scutellarin was selected as the highest concentration. Scutellarin was dissolved in DMSO and initially prepared at 2000 μg·mL^−1^ and then serially two-fold diluted with a medium containing 2% FBS to obtain seven concentration gradients. The final concentration of DMSO in the culture medium was 0.05% (*v*/*v*) in the highest scutellarin concentration group. The diluted scutellarin solutions were added to the 96-well plates at 100 μL per well. The experiment was independently repeated three times as biological replicates (*n* = 3), and each biological replicate included three technical replicate wells. After 24 h of treatment, the medium was removed, and 25 μL MTT solution (Solarbio, Beijing, China) was added to each well. The cells were then incubated at 37 °C for 4 h. The MTT solution was removed, and 150 μL DMSO (Solarbio, Beijing, China) was added to dissolve the formazan crystals. After 30 min of incubation, the optical density (OD) at 490 nm was measured using a microplate reader (Molecular Devices, San Jose, CA, USA).

### 2.4. ZEA Treatment

ZEA (Sigma, St. Louis, MO, USA; catalog No. Z2125) was dissolved in DMSO and stored at −20 °C until use. ZEA was dissolved in DMSO and then diluted with medium containing 2% FBS. Porcine ovarian granulosa cells were seeded into 96-well plates as described above and treated with ZEA at final concentrations of 0, 20, 40, 60, 80, and 100 μM. The final concentration of DMSO in the culture medium did not exceed 0.05% (*v*/*v*) in all ZEA treatment groups. The experiment was independently repeated three times as biological replicates (*n* = 3), and each biological replicate included three technical replicate wells. After 24 h of treatment at 37 °C in a humidified atmosphere containing 5% CO_2_, cell viability was detected using the MTT assay.

### 2.5. Co-Treatment with Scutellarin and ZEA

Porcine ovarian granulosa cells were co-treated with 60 μM ZEA and scutellarin at final concentrations of 2000, 1000, and 500 μg·mL^−1^ for 24 h in a medium with 2% FBS. Untreated cells served as the control group, whereas cells treated with 60 μM ZEA alone were designated as the model group. Scutellarin and ZEA were separately dissolved in DMSO before dilution with culture medium, and the final concentration of DMSO did not exceed 0.1% (*v*/*v*) in all drug-containing treatment groups. The experiment was independently repeated three times as biological replicates (*n* = 3), and each biological replicate included three technical replicate wells. After treatment, the cells were cultured at 37 °C in a humidified atmosphere containing 5% CO_2_, and cell viability was detected using the MTT assay.

### 2.6. Detection of Cell-Cycle Distribution by Flow Cytometry

Porcine ovarian granulosa cells were co-treated with 60 μM ZEA and scutellarin at final concentrations of 2000, 1000, and 500 μg·mL^−1^ for 24 h. Untreated cells served as the control group, whereas cells treated with 60 μM ZEA alone were designated as the model group. Porcine ovarian granulosa cells were independently isolated from three pigs and used as three biological replicates (*n* = 3). After treatment, the cells were collected and fixed in anhydrous ethanol overnight at 4 °C. The cells were then washed with PBS and collected by centrifugation. Subsequently, 500 μL staining working solution (KeyGEN BioTECH, Nanjing, China, RNase A:PI = 1:9) was added to each tube, and the cells were incubated at room temperature for 60 min in the dark. Cell-cycle distribution was analyzed by flow cytometry (BD Biosciences, San Jose, CA, USA) at an excitation wavelength of 488 nm.

### 2.7. RNA Purification and Quantitative Real-Time PCR

Porcine ovarian granulosa cells were seeded into 6-well plates at a density of 1 × 10^6^ cells per well and subjected to co-treatment with scutellarin and ZEA. Total RNA was extracted using TRIzol reagent (Takara, Kusatsu, Japan) according to the manufacturer’s instructions. The extracted RNA was then reverse-transcribed into cDNA using a Takara reverse transcription kit (Takara, Kusatsu, Japan) following the manufacturer’s protocol. Quantitative real-time PCR was performed using an ABI 7500 Real-Time PCR System (Applied Biosystems, Waltham, MA, USA), with *β-actin* used as the internal reference gene. Relative mRNA expression levels were calculated using the 2^−ΔΔCt^ method. The experiment was independently repeated three times as biological replicates (*n* = 3). The primer sequences are listed in Table S1.

### 2.8. Western Blot Analysis

Porcine ovarian granulosa cells were seeded into 6-well plates at a density of 1 × 10^6^ cells per well and subjected to a 24 h co-treatment with scutellarin and ZEA. Total protein was extracted using RIPA lysis buffer (Solarbio, Beijing, China). The protein samples were mixed with 4 × loading buffer (Solarbio, Beijing, China) and denatured at 95 °C for 10 min. Proteins were separated by SDS-PAGE and transferred onto PVDF membranes. The membranes were blocked with 5% skim milk powder prepared in TBST for 2 h and then incubated overnight at 4 °C with primary antibodies against β-actin (1:8000), CDK1 (1:500), CDK2 (1:1000), CDK4 (1:1000), PCNA (1:1000), WNT5A (1:500), β-catenin (1:2000), c-MYC (1:1000), and CCND1 (1:1000) (Proteintech, Wuhan, China). After washing with TBST, the membranes were incubated with horseradish peroxidase-conjugated secondary antibodies at a dilution of 1:20,000 for 1 h. After further washing with TBST, the protein bands were visualized using ECL reagent and imaged with a chemiluminescence imaging system (Bio-Rad Laboratories, Hercules, CA, USA). Band intensities were quantified using ImageJ 1.53e software. The experiment was independently repeated three times as biological replicates (*n* = 3).

### 2.9. Effect of WNT5A on Cell-Cycle-Related Proteins in Porcine Ovarian Granulosa Cells

Two siRNA sequences targeting the porcine *WNT5A* gene were synthesized by Qingke Biotechnology and designated siWNT5A-1 and siWNT5A-2. The negative control sequence was designated as siRNA-NC, as shown in Table S2. Porcine ovarian granulosa cells were seeded into 6-well plates at a density of 1 × 10^6^ cells/mL. When the cells reached approximately 40% confluence, they were transfected with siWNT5A using Lipofectamine™ 2000 reagent (Thermo Fisher Scientific, Waltham, MA, USA) according to the manufacturer’s instructions. WNT5A knockdown efficiency was evaluated by qRT-PCR and Western blot. The siRNA sequence with the highest knockdown efficiency was selected for subsequent experiments.

The cells were divided into the following groups: non-interference control group (siRNA-NC), WNT5A knockdown group (siWNT5A), ZEA-treated non-interference group (siRNA-NC + ZEA), ZEA-treated WNT5A knockdown group (siWNT5A + ZEA), ZEA and scutellarin (Scu) co-treated non-interference groups (siRNA-NC + ZEA + Scu 2000, siRNA-NC + ZEA + Scu 1000, and siRNA-NC + ZEA + Scu 500), and ZEA and scutellarin co-treated WNT5A knockdown groups (siWNT5A + ZEA + Scu 2000, siWNT5A + ZEA + Scu 1000, and siWNT5A + ZEA + Scu 500). All groups were treated for 24 h. The cells were then collected, and total protein was extracted to measure CDK1, CDK2, CDK4, and PCNA expression by Western blot. The experiment was independently repeated three times as biological replicates (*n* = 3).

### 2.10. Statistical Analysis

Statistical analysis was performed using GraphPad Prism 8 software. All data are presented as mean ± SEM. Comparisons among groups were conducted using one-way analysis of variance (ANOVA). *p* < 0.05 was considered statistically significant, whereas *p* > 0.05 was considered not significant (* *p* < 0.05, ** *p* < 0.01, *** *p* < 0.001, **** *p* < 0.0001).

## 3. Results

### 3.1. Isolation and Identification of Porcine Ovarian Granulosa Cells

**Figure 1:**
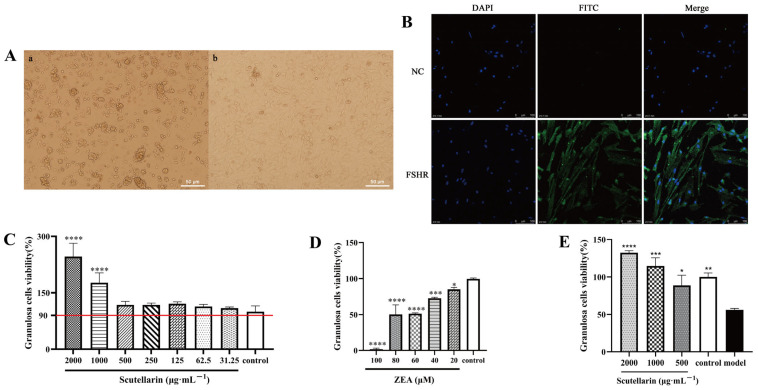
Isolation and identification of porcine ovarian granulosa cells and evaluation of scutellarin protection against ZEA-induced injury. (**A**) Morphology of porcine ovarian granulosa cells. (**a**) Cells showing polygonal morphology after 48 h of culture. (**b**) Cells exhibiting spindle-shaped, elongated, and irregular triangular morphologies at 5 days post-culture. Scale bar = 50 μm. (**B**) Identification of porcine ovarian granulosa cells by FSHR immunofluorescence. Blue fluorescence indicates DAPI-stained nuclei, and green fluorescence indicates FSHR expression. NC, negative control without primary antibody. Scale bar = 100 μm. (**C**) Effects of different concentrations of scutellarin on the viability of porcine ovarian granulosa cells. The red reference line indicates 90% cell viability. Control, untreated cells. (**D**) Effects of different concentrations of ZEA on the viability of porcine ovarian granulosa cells. Control, untreated cells. (**E**) Viability of porcine ovarian granulosa cells following co-treatment with different concentrations of scutellarin and 60 μM ZEA. Control, untreated cells; Model, cells treated with 60 μM ZEA alone. Data are presented as mean ± SEM. (*n* = 3, **** *p* < 0.0001, *** *p* < 0.001, ** *p* < 0.01, * *p* < 0.05).

Porcine ovarian granulosa cells were isolated and cultured at 37 °C in a humidified atmosphere containing 5% CO_2_. After 48 h of culture, the cells exhibited a polygonal morphology under microscopic observation (Figure 1(Aa)). After 5 days of culture, the cells reached more than 90% confluence and displayed spindle-shaped, elongated, and irregularly triangular morphologies (Figure 1(Ab)).

Under confocal laser microscopy, blue fluorescence indicated DAPI-stained nuclei, whereas green fluorescence indicated positive FSHR staining. No green fluorescence was observed in the blank control group, whereas clear green fluorescence was detected in the FSHR-positive group (Figure 1B). These results confirm that the isolated and cultured cells were porcine ovarian granulosa cells and were suitable for subsequent experiments.

### 3.2. Effect of Scutellarin on the Viability of Porcine Granulosa Cells

After treatment with different concentrations of scutellarin for 24 h, the MTT-derived viability values of porcine ovarian granulosa cells remained above 90% at all tested concentrations ranging from 31.25 to 2000 μg·mL^−1^ compared with the control group (Figure 1C). Notably, treatment with 1000 and 2000 μg·mL^−1^ scutellarin resulted in significantly higher cell viability than the control group (*p* < 0.0001). These results indicate that scutellarin exhibits no obvious cytotoxic effects on porcine ovarian granulosa cells within the tested concentration range.

### 3.3. Establishment of a ZEA-Induced Injury Model in Porcine Ovarian Granulosa Cells

After being treated with different concentrations of ZEA for 24 h, cell viability was significantly reduced in the 20, 40, 60, 80, and 100 μM groups compared with the control group (*p* < 0.05) (Figure 1D). In particular, 60 μM ZEA reduced cell viability by approximately 50%. Therefore, 60 μM ZEA was selected to establish the porcine ovarian granulosa cell injury model for subsequent experiments, as it produced a moderate injury condition while retaining a sufficient number of viable cells for intervention analysis.

### 3.4. Effect of Scutellarin on Cell Viability in the ZEA-Induced Injury Model of Porcine Ovarian Granulosa Cells

Porcine ovarian granulosa cells were treated with 60 μM ZEA in combination with 2000, 1000, or 500 μg·mL^−1^ scutellarin for 24 h. Untreated cells served as the control group, whereas cells treated with 60 μM ZEA alone were designated as the model group. Compared with the model group, co-treatment with 2000 and 1000 μg·mL^−1^ scutellarin significantly increased cell viability to 132% and 115%, respectively. In the 500 μg·mL^−1^ scutellarin co-treatment group, cell viability was 89% (Figure 1E). These results suggest that scutellarin attenuates the ZEA-induced reduction in cell viability in porcine ovarian granulosa cells.

### 3.5. Effects of Scutellarin and ZEA on the Cell-Cycle Distribution of Porcine Ovarian Granulosa Cells

Flow cytometry was used to analyze cell-cycle distribution in porcine ovarian granulosa cells after 24 h treatment with ZEA and scutellarin. The proportion of cells in the G0/G1 phase was significantly lower in the model group than in the control group (*p* < 0.001). Compared with the model group, treatment with 2000, 1000, and 500 μg·mL^−1^ scutellarin significantly increased the proportion of cells in the G0/G1 phase (*p* < 0.01).

**Figure 2:**
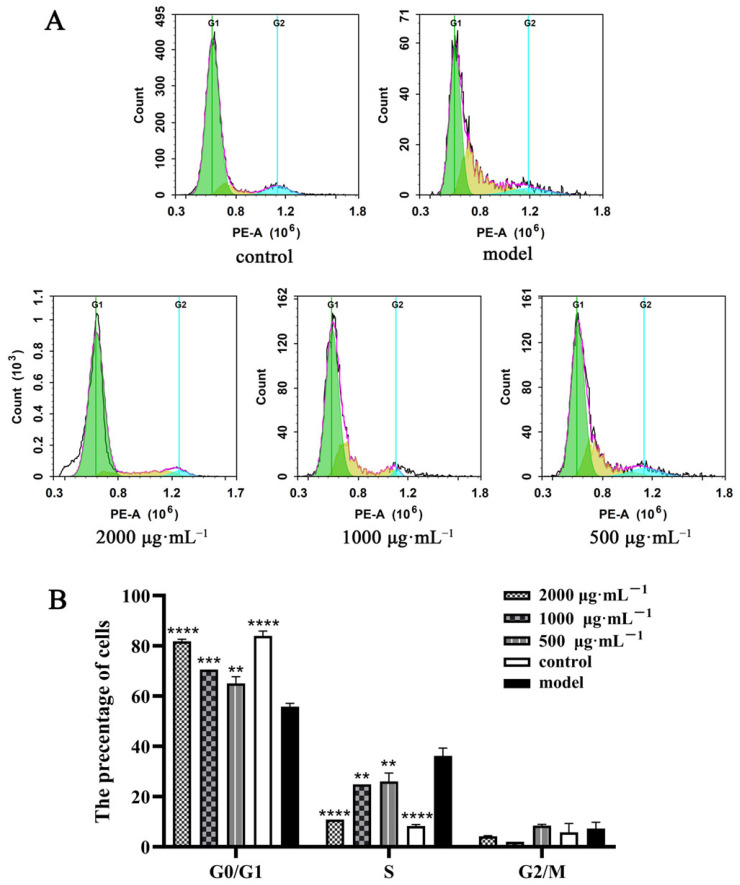
Effects of scutellarin and ZEA co-treatment on cell-cycle distribution in porcine ovarian granulosa cells. (**A**) Cell-cycle distribution was detected by flow cytometry. Green, yellow, and cyan areas represent the G0/G1, S, and G2/M phases, respectively. (**B**) Quantitative analysis of the cell-cycle phase proportions. Control, untreated cells; Model, cells treated with 60 μM ZEA alone. (*n* = 3, **** *p* < 0.0001, *** *p* < 0.001, ** *p* < 0.01).

Conversely, the proportion of cells in the S phase was significantly higher in the model group than in the control group (*p* < 0.001). Compared with the model group, treatment with 2000, 1000, and 500 μg·mL^−1^ scutellarin significantly reduced the proportion of cells in the S phase (*p* < 0.01). No significant differences in the G2/M phase proportion were observed among the groups (*p* > 0.05) (Figure 2B). These results indicate that ZEA induced cell-cycle disturbance characterized by S-phase accumulation in porcine ovarian granulosa cells, whereas scutellarin restored the disrupted cell-cycle distribution.

### 3.6. Effect of Scutellarin on the Expression of Cell-Cycle-Related Genes and Proteins in ZEA-Treated Porcine Ovarian Granulosa Cells

**Figure 3:**
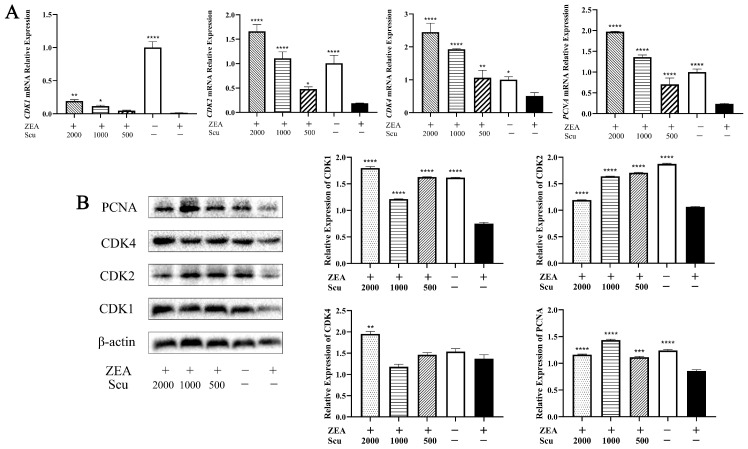
Effects of scutellarin and ZEA co-treatment on mRNA and protein expression of cell-cycle-related factors in porcine ovarian granulosa cells. (**A**) mRNA expression levels of cell-cycle-related genes detected by qRT-PCR. (**B**) Protein expression levels of cell-cycle-related proteins detected by Western blot. (*n* = 3, **** *p* < 0.0001, *** *p* < 0.001, ** *p* < 0.01, * *p* < 0.05).

qRT-PCR was performed to measure the mRNA expression levels of cell-cycle-related genes *CDK1*, *CDK2*, *CDK4*, and *PCNA* in porcine ovarian granulosa cells after 24 h of co-treatment with scutellarin and ZEA. As shown in Figure 3A, compared with the control group, the model group showed significantly decreased mRNA expression of *CDK1*, *CDK2*, *CDK4*, and *PCNA* (*p* < 0.05). Compared with the model group, co-treatment with 2000 μg·mL^−1^ scutellarin significantly increased the mRNA expression of *CDK1*, *CDK2*, *CDK4*, and *PCNA* (*p* < 0.01); co-treatment with 1000 μg·mL^−1^ scutellarin also significantly increased the mRNA expression of these genes (*p* < 0.05). In the 500 μg·mL^−1^ scutellarin group, the mRNA expression of *CDK2*, *CDK4*, and *PCNA* was significantly increased compared with the model group (*p* < 0.05).

Western blot analysis was used to evaluate the corresponding protein expression levels (Figure 3B). Compared with the control group, the model group showed significantly reduced protein expression of CDK1, CDK2, and PCNA (*p* < 0.0001). Compared with the model group, all scutellarin co-treatment groups showed significantly increased CDK1 and CDK2 protein expression (*p* < 0.0001). CDK4 protein expression was significantly elevated only in the 2000 μg·mL^−1^ scutellarin group (*p* < 0.01), whereas PCNA protein expression was significantly increased in all scutellarin co-treatment groups (*p* < 0.001). These results indicate that scutellarin attenuated the ZEA-induced downregulation of cell-cycle-related genes and proteins in porcine ovarian granulosa cells.

### 3.7. Effects of Scutellarin on the Expression of Wnt/β-Catenin Signaling-Related Proteins in ZEA-Treated Porcine Ovarian Granulosa Cells

**Figure 4:**
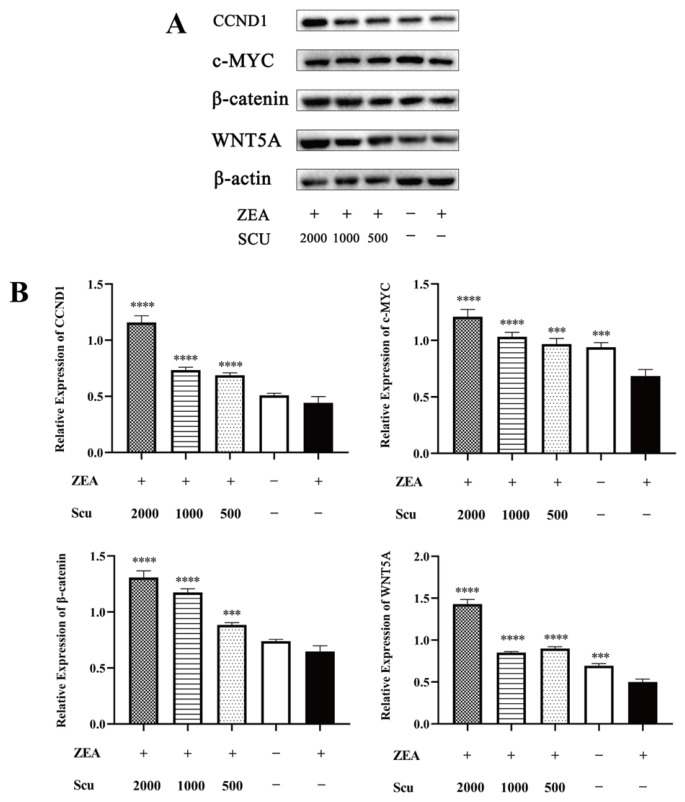
Effects of scutellarin and ZEA co-treatment on the expression of Wnt/β-catenin signaling-related proteins in porcine ovarian granulosa cells. (**A**) Representative Western blot images of WNT5A, β-catenin, c-MYC, and CCND1; β-actin was used as a loading control. (**B**) Quantitative analysis of protein expression levels of WNT5A, β-catenin, c-MYC, and CCND1. (*n* = 3, **** *p* < 0.0001, *** *p* < 0.001).

Western blot was used to detect the protein expression levels of WNT5A, β-catenin, c-MYC, and CCND1 in porcine ovarian granulosa cells after 24 h of co-treatment with scutellarin and ZEA. As shown in Figure 4, compared with the control group, the model group showed significantly decreased expression of WNT5A and c-MYC (*p* < 0.001), whereas β-catenin and CCND1 expression did not differ significantly (*p* > 0.05). Compared with the model group, scutellarin co-treatment significantly increased the expression of WNT5A, β-catenin, c-MYC, and CCND1 (*p* < 0.001). These results indicate that scutellarin upregulated the expression of Wnt/β-catenin signaling-related proteins and, in particular, restored the ZEA-induced downregulation of WNT5A and c-MYC.

### 3.8. WNT5A Knockdown Attenuates Scutellarin-Mediated Regulation of Cell-Cycle-Related Proteins in Porcine Ovarian Granulosa Cells

**Figure 5:**
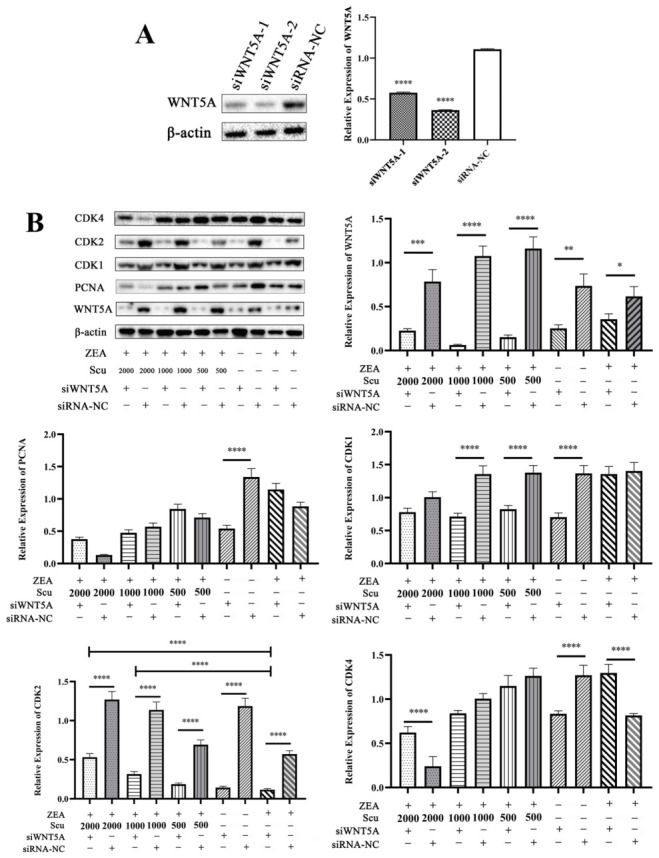
WNT5A is involved in scutellarin-mediated regulation of cell-cycle-related proteins in porcine ovarian granulosa cells. (**A**) Identification of siRNA-mediated WNT5A knockdown efficiency. (**B**) Effect of siRNA-mediated WNT5A knockdown on the protein expression of CDK1, CDK2, CDK4, and PCNA. (*n* = 3, **** *p* < 0.0001, *** *p* < 0.001, ** *p* < 0.01, * *p* < 0.05).

To determine whether WNT5A is involved in scutellarin-mediated regulation of cell-cycle-related proteins, two siRNA sequences targeting *WNT5A* were designed and synthesized. Knockdown efficiency was evaluated by Western blot. As shown in Figure 5A, both siWNT5A-1 and siWNT5A-2 significantly reduced WNT5A protein expression compared with the siRNA-NC group (*p* < 0.0001). Since siWNT5A-2 demonstrated higher knockdown efficiency than siWNT5A-1, it was selected for subsequent experiments.

Porcine ovarian granulosa cells were then transfected with siWNT5A-2 for 48 h, followed by 24 h co-treatment with ZEA and scutellarin. The protein expression levels of CDK1, CDK2, CDK4, and PCNA were detected by Western blot (Figure 5B). Compared with the siRNA-NC group, the siWNT5A group showed significantly decreased expression of PCNA, CDK1, CDK2, and CDK4 (*p* < 0.0001), indicating that WNT5A knockdown affected the expression of these cell-cycle-related proteins.

Compared with the siRNA-NC + ZEA + Scu group, the siWNT5A + ZEA + Scu group exhibited significantly lower WNT5A protein expression (*p* < 0.001). No significant difference in WNT5A expression was observed between the siWNT5A + ZEA group and the siWNT5A + ZEA + Scu group (*p* > 0.05), suggesting that scutellarin did not restore WNT5A expression after WNT5A knockdown. Compared with the siWNT5A + ZEA group, the siWNT5A + ZEA + Scu group showed no significant changes in PCNA, CDK1, or CDK4 expression (*p* > 0.05). However, CDK2 expression was significantly increased in the siWNT5A + ZEA + Scu 2000 μg·mL^−1^ and siWNT5A + ZEA + Scu 1000 μg·mL^−1^ groups (*p* < 0.0001). These results indicate that scutellarin regulates PCNA, CDK1, and CDK4 expression in a WNT5A-dependent manner, whereas its effect on CDK2 expression may occur through a WNT5A-independent mechanism.

## 4. Discussion

Because granulosa cell dysfunction is closely associated with ZEA-induced reproductive injury, we first established and characterized a primary porcine granulosa cell model [22,23]. The evaluation of different concentrations of scutellarin showed that scutellarin did not exert obvious cytotoxic effects on porcine ovarian granulosa cells within the tested concentration range of 31.25–2000 μg·mL^−1^. In the ZEA-induced injury model, 60 μM ZEA reduced cell viability to approximately 50%. This concentration was selected based on preliminary dose–response experiments as it approximated the IC50 value, which is a common strategy to establish ZEA cytotoxicity models for mechanistic studies [9,24]. However, 60 μM ZEA represents an in vitro toxicological challenge rather than physiological exposure, and the findings should not be directly extrapolated to in vivo conditions [6,12]. Co-treatment with scutellarin significantly restored cell viability compared with the ZEA model group, particularly at 1000 and 2000 μg·mL^−1^, suggesting that scutellarin may alleviate ZEA-induced cellular injury in porcine ovarian granulosa cells.

Given the improvement in MTT-derived viability observed after scutellarin co-treatment, we further investigated whether this effect was associated with cell-cycle regulation. Flow cytometry analysis showed that ZEA treatment altered the cell-cycle distribution of porcine ovarian granulosa cells, as reflected by increased S-phase and reduced G0/G1 population. Previous studies have reported that ZEA promotes the transition of mouse KK-1 granulosa cells from G1 to S phase, ultimately resulting in S-phase arrest [25]. Our results are generally consistent with these findings. However, this comparison should be interpreted cautiously, as KK-1 is an immortalized mouse granulosa cell line, whereas the present study used primary granulosa cells isolated from porcine ovaries. Moreover, species-dependent differences in ZEA sensitivity have been reported in domestic and laboratory animals [6,26], further supporting the need to validate ZEA-induced cell-cycle responses in porcine primary granulosa cells. Thus, the present study provides direct evidence of ZEA-induced cell-cycle disturbance in primary porcine granulosa cells, mainly characterized by S-phase accumulation in porcine primary granulosa cells. Scutellarin treatment significantly reduced the proportion of cells in S phase and increased the population in G0/G1 phase, suggesting that it may alleviate ZEA-induced cell-cycle disturbance. Notably, no significant change was observed in the G2/M phase among the treatment groups, indicating that the cell-cycle disturbance in this model was mainly characterized by S-phase accumulation rather than generalized redistribution across all cell-cycle phases.

Cell-cycle progression is regulated by multiple signaling pathways and cell-cycle-related proteins. Scutellarin has been reported to promote proliferation in human osteoblasts by promoting p65 phosphorylation and upregulating CXCR4 expression [27]. In bovine large follicle granulosa cells, knockdown of IL-11Rα induced G1 phase arrest, downregulated PCNA and CCND1 expression, and activated the JAK1/STAT3 signaling pathway [28]. CDKs are key positive regulators of the cell-cycle progression, while PCNA participates in DNA replication and repair and is commonly used as an indicator of cell-cycle progression and proliferative activity [29]. In addition, ochratoxin A has been shown to induce S-phase arrest and apoptosis in human embryonic kidney cells by downregulating Cyclin A2 and CDK2, whereas simvastatin mediates S-phase arrest in multiple myeloma cells through the Chk1-cdc25A-cyclinA/CDK2 pathway [30,31]. In this study, ZEA decreased the expression of CDK1, CDK2, CDK4, and PCNA, while scutellarin co-treatment significantly reversed these changes. Combined with the flow cytometry results, these findings suggest that scutellarin may alleviate ZEA-induced cell-cycle disruption by restoring the expression of cell-cycle-related factors.

WNT5A is involved in ovarian follicular development and granulosa cell function. Granulosa cell-specific knockout of WNT5A has been reported to increase follicular atresia, reduce ovulation rate, and downregulate FSH-responsive genes, with possible involvement of the β-catenin canonical pathway [32]. β-catenin also contributes to granulosa cell proliferation and normal ovarian function, as abnormal β-catenin signaling in granulosa cells can disrupt follicular structure and impair fertility [33]. Scutellarin has been reported to inhibit gastric cancer proliferation, ameliorate colitis-associated colorectal cancer, and improve cartilage degeneration in osteoarthritis via the Wnt/β-catenin pathway [34,35,36]. Our previous studies suggested that WNT5A may be associated with the protective effect of scutellarin against ZEA-induced granulosa cell injury [18]. Based on this rationale, the present study further examined the expression of Wnt/β-catenin signaling-related proteins under ZEA exposure. Scutellarin significantly upregulated the expression of WNT5A, β-catenin, c-MYC, and CCND1, suggesting that these proteins may participate in the response to scutellarin in ZEA-treated porcine granulosa cells. However, further functional validation of β-catenin signaling activity is needed to confirm whether the canonical Wnt/β-catenin pathway is activated.

To clarify whether scutellarin promotes cell-cycle progression via the WNT5A pathway, we used siRNA to knock down WNT5A expression. Among the two interference sequences tested (siWNT5A-1 and siWNT5A-2), siWNT5A-2 showed higher knockdown efficiency and was selected for subsequent experiments. After WNT5A knockdown, scutellarin failed to restore WNT5A expression in porcine ovarian granulosa cells. This finding is generally consistent with our previous observations in mouse granulosa cells [18], suggesting that WNT5A-associated regulation may be shared, at least in part, between mouse and porcine granulosa cells. Nevertheless, further cross-species studies are needed to determine the extent to which this mechanism is conserved.

Furthermore, WNT5A knockdown affected the expression of PCNA, CDK1, CDK2, and CDK4. However, scutellarin still significantly increased CDK2 expression after WNT5A knockdown, suggesting that CDK2 is not a strictly WNT5A-dependent downstream factor in this model. CDK2 activity and expression can be influenced by multiple upstream regulators, including cyclin E/cyclin A availability, DNA damage checkpoint signaling, and oxidative stress-related pathways [37,38,39]. Because ZEA can induce oxidative stress and DNA damage-related responses in granulosa cells [11,40], scutellarin may modulate CDK2 through additional WNT5A-independent pathways. In contrast, the upregulation of PCNA, CDK1, and CDK4 was largely abolished following WNT5A knockdown, indicating that scutellarin regulates the expression of these proteins in a WNT5A-dependent manner. In our previous study, WNT5A knockdown attenuated the anti-apoptotic effect of scutellarin against ZEA, and SPR analysis further demonstrated a direct interaction between scutellarin and WNT5A [18]. Taken together, these findings suggest that WNT5A is involved in scutellarin-mediated restoration of CDK1, CDK4, and PCNA expression in ZEA-treated porcine ovarian granulosa cells, whereas CDK2 may be regulated through WNT5A-independent mechanisms. Future studies should perform WNT5A rescue experiments to determine whether restoring WNT5A activity is sufficient to counteract ZEA-induced granulosa cell injury.

## 5. Conclusions

In summary, scutellarin attenuated ZEA-induced cell-cycle disturbance in porcine ovarian granulosa cells. Mechanistically, scutellarin increased the expression of Wnt/β-catenin signaling-related proteins and regulated cell-cycle-related factors. WNT5A knockdown largely abolished the effects of scutellarin on PCNA, CDK1, and CDK4 expression, suggesting that WNT5A is involved in scutellarin-mediated regulation of these proteins. These findings indicate that scutellarin may alleviate ZEA-induced granulosa cell injury partly through WNT5A-associated regulation of cell-cycle-related proteins.

## Data Availability

The original contributions presented in this study are included in the article/Supplementary Materials. Further inquiries can be directed to the corresponding author.

## Supplementary Materials

The following supporting information can be downloaded at https://www.mdpi.com/article/10.3390/vetsci13070719/s1; Table S1: Primer sequences. Table S2: siRNA sequence. Figure S1. Original uncropped Western blot images for Figure 3B. Figure S2. Original uncropped Western blot images for Figure 4A. Figure S3. Original uncropped Western blot images for Figure 5A. Figure S4. Original uncropped Western blot images for Figure 5B.

## Abbreviations

The following abbreviations are used in this manuscript:

ZEA	Zearalenone
FSHR	Follicle-stimulating hormone receptor
CDK1	Cyclin-dependent kinase 1
CDK2	Cyclin-dependent kinase 2
CDK4	Cyclin-dependent kinase 4
PCNA	Proliferating cell nuclear antigen
WNT5A	Protein Wnt-5a
β-catenin	Catenin beta 1
c-MYC	Myc proto-oncogene protein
CCND1	G1/S-specific cyclin-D1
NRF2	Nuclear factor erythroid2-related factor 2
Scu	Scutellarin
CXCR4	C-X-C chemokine receptor type 4
IL-11Rα	Interleukin-11 receptor subunit alpha
β-actin	Beta-actin
