# Supplementary material for: Scutellarin Alleviates Zearalenone-Induced Injury in Porcine Ovarian Granulosa Cells Through WNT5A-Associated Regulation of Cell-Cycle-Related Proteins
Source: Vet Sci. 2026 Jul 22;13(7):719. doi: 10.3390/vetsci13070719 (PMC13431581; doi:10.3390/vetsci13070719)
Supplement: Supplementary file 1 [file vetsci-13-00719-s001.zip › Supplementary Figures.pdf]

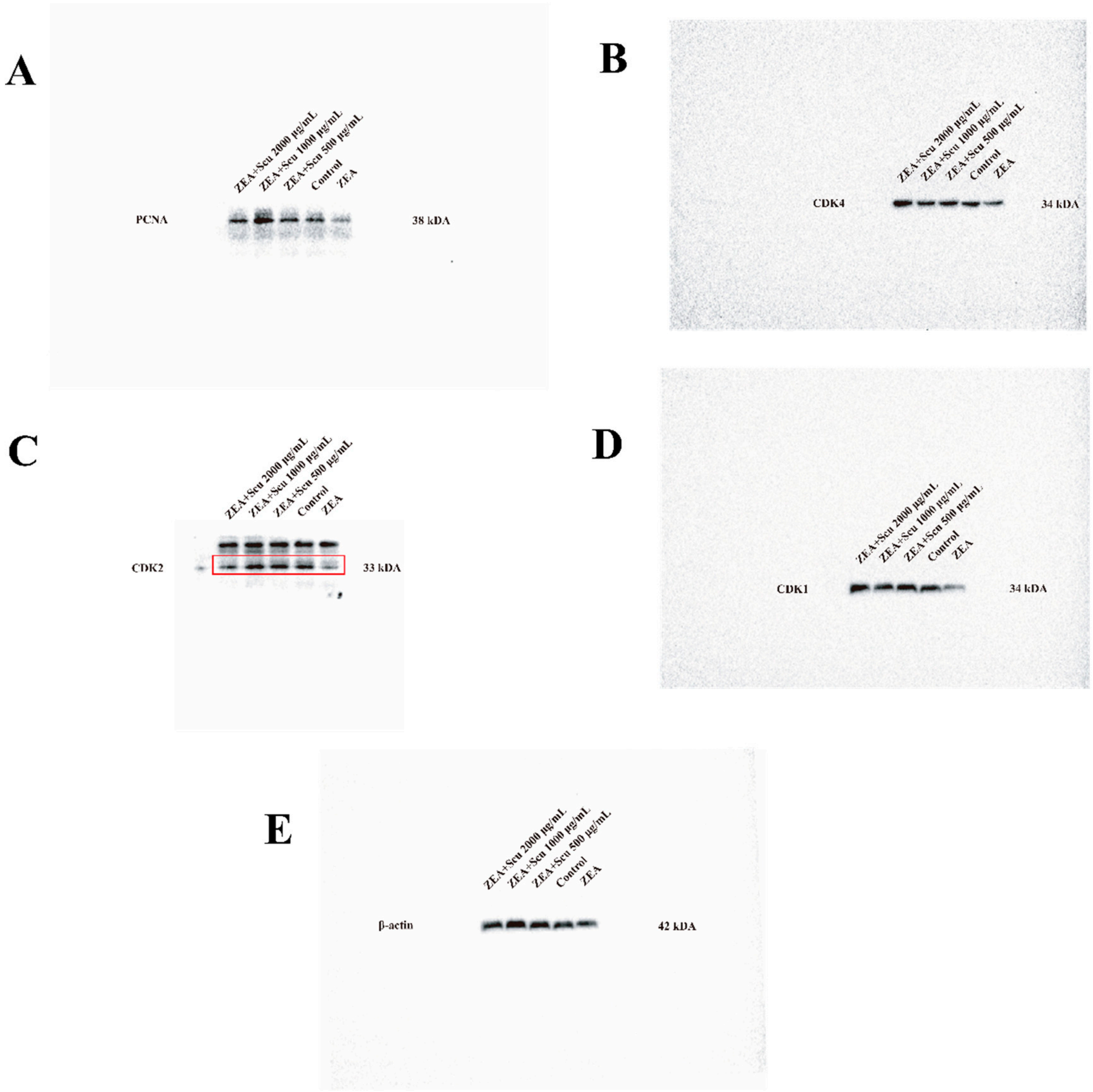

Figure S1. Original uncropped Western blot images for Figure 3B.
(A) PCNA; (B) CDK4; (C) CDK2; (D) CDK1; (E) β-actin.

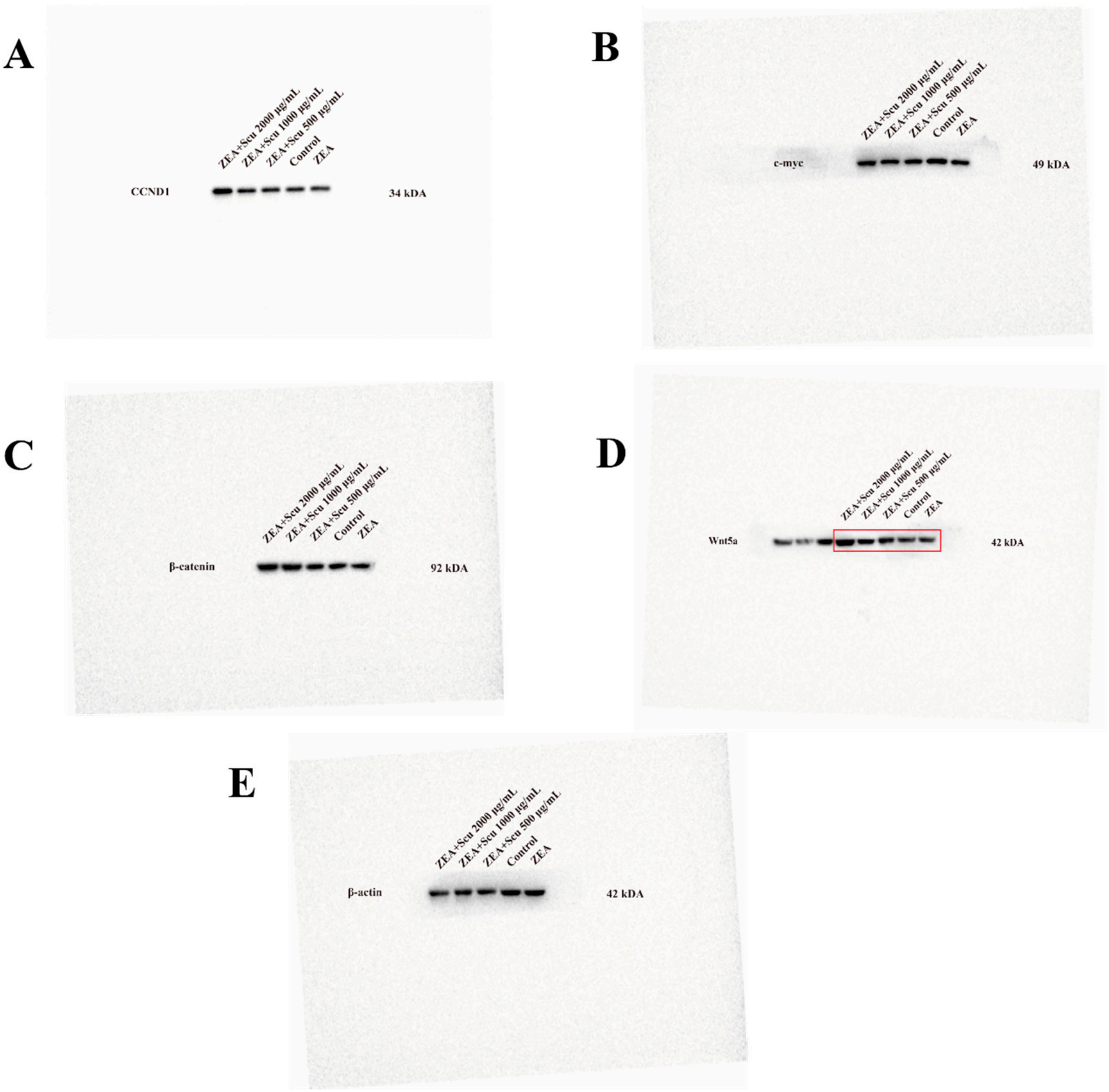

Figure S2. Original uncropped Western blot images for Figure 4A.
(A) CCND1; (B) c-MYC; (C) β-catenin; (D) WNT5A; (E) β-actin.

A

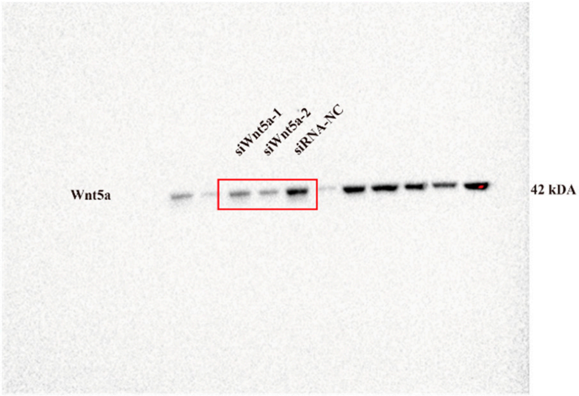

B

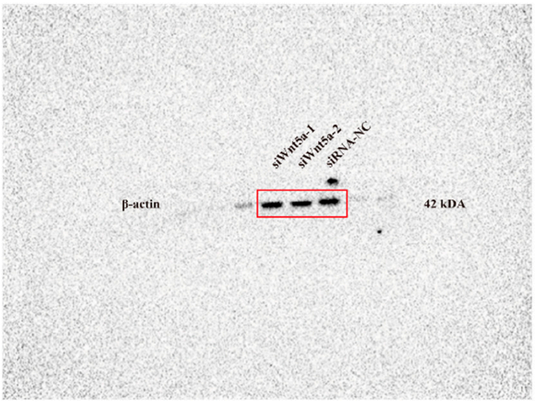

Figure S3. Original uncropped Western blot images for Figure 5A.
(A) WNT5A; (B) β-actin.

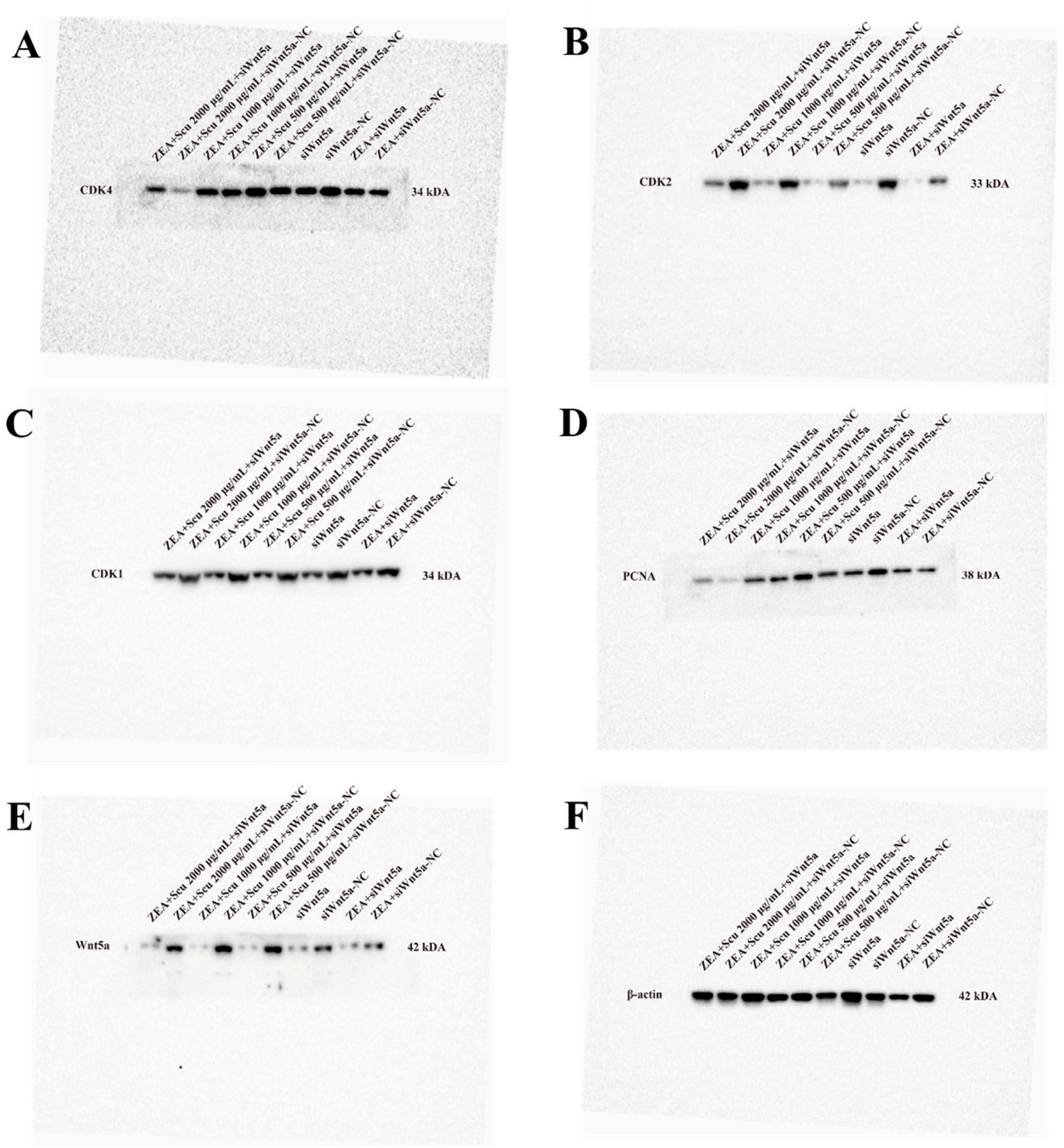

Figure S4. Original uncropped Western blot images for Figure 5B.

(A) CDK4; (B) CDK2; (C) CDK1; (D) PCNA; (E) WNT5A; (F) β-actin.
